# A robust statistical approach for finding informative spatially associated pathways

**DOI:** 10.1093/bib/bbae543

**Published:** 2024-10-25

**Authors:** Leqi Tian, Jiashun Xiao, Tianwei Yu

**Affiliations:** School of Data Science, The Chinese University of Hong Kong, Shenzhen (CUHK-Shenzhen), Shenzhen, Guangdong 518172, P.R. China; Shenzhen Research Institute of Big Data, Shenzhen, Guangdong 518172, P.R. China; Shenzhen Research Institute of Big Data, Shenzhen, Guangdong 518172, P.R. China; School of Data Science, The Chinese University of Hong Kong, Shenzhen (CUHK-Shenzhen), Shenzhen, Guangdong 518172, P.R. China; Shenzhen Research Institute of Big Data, Shenzhen, Guangdong 518172, P.R. China

**Keywords:** spatial transcriptomic, spatial variability, functional pathways, statistical testing, tissue architecture

## Abstract

Spatial transcriptomics offers deep insights into cellular functional localization and communication by mapping gene expression to spatial locations. Traditional approaches that focus on selecting spatially variable genes often overlook the complexity of biological pathways and the interactions among genes. Here, we introduce a novel framework that shifts the focus towards directly identifying functional pathways associated with spatial variability by adapting the Brownian distance covariance test in an innovative manner to explore the heterogeneity of biological functions over space. Unlike most other methods, this statistical testing approach is free of gene selection and parameter selection and allows nonlinear and complex dependencies. It allows for a deeper understanding of how cells coordinate their activities across different spatial domains through biological pathways. By analyzing real human and mouse datasets, the method found significant pathways that were associated with spatial variation, as well as different pathway patterns among inner- and edge-cancer regions. This innovative framework offers a new perspective on analyzing spatial transcriptomic data, contributing to our understanding of tissue architecture and disease pathology. The implementation is publicly available at https://github.com/tianlq-prog/STpathway.

## Introduction

The rapid developments in Spatially Resolved Transcriptomics technology have provided omics research with an unprecedented perspective. The technology can simultaneously capture the expression of genes along with their spatial coordinates. This combination effectively bridged the gap between transcriptomics and histological topology [1, 2]. Technologies like Visium integrate microfluidic partitioning with barcoded bead arrays to analyze $\sim $5000 spots with a diameter of 55 $\mu $m each, offering a detailed glimpse into the spatial variation of gene expression. Slide-seq transfers RNA from tissue sections onto a surface arrayed with DNA-barcoded beads measuring $\sim $10 $\mu $m, enabling the sequencing of RNA from thousands of spatially resolved points within the tissue [3]. High-Definition Spatial Transcriptomics can provide sub-cellular insights, capturing the heterogeneity of tissue architecture and the nuanced shifts in gene expression patterns with remarkable clarity [4].

Using spatial transcriptomics data, researchers can utilize tissue spatial location when studying gene expression patterns [3]. Combined with spatial coordinates, researchers can explore how gene expression changes dynamically along with the cellular microenvironment, enabling the discovery of new knowledge on cellular regulation and communication. When analyzing gene expression patterns, identifying highly variable genes [5] is a straightforward approach that has been widely used in single-cell RNA-seq (scRNA-seq) data analysis. By incorporating spatial information, spatially variable genes (SVGs) are defined as genes showing differential expression in different tissue regions. Studying their involvement in various biological processes reveals the structural and functional organization of the tissue [2]. A number of methodologies for detecting SVGs have been introduced in recent years. For example, SpatialDE distinguishes gene variations as spatial or non-spatial by fitting each gene with a Gaussian process model [6]. NnSVG estimates the spatial association of each gene by employing nearest neighbor Gaussian processes within the spatial covariance function [7]. SPARK-X, using a non-parametric approach, assesses the covariance similarity between the expression matrix of each gene and the spatial coordinates matrix to conduct hypothesis testing [8]. Big-small patch identifies SVGs by comparing variances in gene expression across two spatial granularities [9]. In addition, there are several other methods, including Trendsceek [10] and MERINGUE [11], $etc$.

While gene-centric analyses shed light on the molecular mechanisms linked to specific cellular states, they often miss the complexity of biological pathways and biological network dynamics. Changes in spatial gene expression patterns should reflect coordinated biological process activities and interactions. For non-spatial omics data, many previous works have exploited pathway information for improved data inperpretation. For example, PASNet integrates pathway information into sparse deep neural networks to predict the long-term survival of patients with glioblastoma multiforme (GBM) [12]. At the same time, PathCNN applies convolutional neural networks to biological pathway images to Predict GBM [13]. Integration of biological pathway data not only improves model performance but also enhances the model’s biological interpretability. Therefore, we aim at going beyond gene-level analysis and use biological pathway and gene network perspectives to explain spatial expression properties.

Here we introduce a novel framework that focuses on identifying functional pathways associated with spatial location to explore the heterogeneity of biological functions over space. By concentrating on pathways, our approach delves deeper into the spatial heterogeneity of biological functions and uncovers how cells coordinate their activities through biological processes across different spatial domains. Given the complexity of pathway activity, we go beyond linear association by employing the Brownian distance correlation (dCor) test in a novel set-up to evaluate the dependency of two random vectors with different dimensionality. The test is free of parameter selection. The test can find informative spatially varying pathways from two aspects: (i) finding significant pathways associated with spatial location, and (ii) finding different pathway patterns between pre-specified spatial areas, such as the inner- and edge-cancer regions.

## Methods

Spatial transcriptomics data involve both gene expression counts and spatial position information. We define the gene expression matrix as $G \in \mathbb{R}^{n \times r}$ and spatial location matrix as $L \in \mathbb{R}^{n \times 2}$, where $n$ is the number of sequencing locations, and $r$ is the number of genes.

### Covariance coefficient and Brownian distance covariance test

#### Covariance coefficient

Independence is crucial in various applications to infer whether two variables depend on each other. The most classical measure of dependence is the Pearson correlation coefficient [14], which is defined as follows:


\begin{align*} & r=\frac{\sum\left(X_i-\bar{X}\right)\left(Y_i-\bar{Y}\right)}{\sqrt{\sum\left(X_i-\bar{X}\right)^2 \sum\left(Y_i-\bar{Y}\right)^2}}, \end{align*}

where $X_{i}$ and $Y_{i}$ are the individual sample points, and $\bar{X}$ and $\bar{Y}$ are the means of $X$ and $Y$, respectively. The Pearson correlation coefficient measure linear dependence, and in the bivariate normal case $r=0$ is equivalent to independence. Another widely used correlation coefficient is the Spearman correlation coefficient, a non-parametric measure of rank correlation [15]. The Spearman correlation coefficient is calculated by converting the data into ranks and then applying the Pearson correlation formula to these ranks:


\begin{align*} & r_s=1-\frac{6 \sum d_i^2}{n\left(n^2-1\right)} \end{align*}

where $d_{i} = \text{rank}(X_{i}) - \text{rank}(Y_{i})$ is the difference between the ranks of each observation, and $n$ is the number of observations. This correlation can access monotonic relationships. Although these two correlation coefficients are widely applied in fields such as clinical trials and financial data analysis, they have the limitation that they cannot characterize nonlinear or nonmonotone dependence. Furthermore, a correlation of zero does not imply independence in most cases. To fulfill the gap, dCor has been proposed as an entirely new approach to address these limitations [16].

#### Brownian distance covariance test

The concept of distance covariance (dCov) was introduced as a metric to measure the dependence between two random vectors $X$ and $Y$ of arbitrary dimensions [16–18]. Unlike the Pearson correlation, dCor can detect both linear and nonlinear associations and is flexible with respect to dimensionality. The dCor $R$, a standardized version of the dCov $V$, satisfies $0\leq R \leq 1$ and is designed to have the two properties:

1. $R(X,Y)$ is defined for $X$ and $Y$ in arbitrary dimension.

2. $R(X,Y) = 0$ can characterizes independence of $X$ and $Y$.

Let us consider two random vectors $X \in \mathbb{R}^{p}$ and $Y \in \mathbb{R}^{q}$, where $p$ and $q$ are positive integers. The characteristic functions of $X$ and $Y$ are represented as $f_{X}$ and $f_{Y}$, with their joint characteristic function denoted by $f_{X,Y}$. The discrepancy between $f_{X,Y}$ and $f_{X} f_{Y}$ can be quantified by the distance $\left \|f_{X, Y}-f_{X} f_{Y}\right \|$. Thus, we can test the independence using the hypothesis as the following:


\begin{align*} &H_0: f_{X, Y}=f_X f_Y \quad \text{v.s} \quad H_1: f_{X, Y} \neq f_X f_Y.\end{align*}

The Distance covariance $V$ is designed as a measure of dependence, which is defined by


(1) \begin{align*}& \begin{aligned} \mathcal{V}^{2}(X, Y ; w) & =\left\|f_{X, Y}(t, s)-f_{X}(t) f_{Y}(s)\right\|_{w}^{2} \\ & =\int_{\mathbb{R}^{p+q}}\left|f_{X, Y}(t, s)-f_{X}(t) f_{Y}(s)\right|{}^{2} w(t, s) d t d s, \end{aligned}\end{align*}

with weight function $w(t,s)$. The Distance covariance $V$ has an important property that $V^{2}(X,Y;w) =0$ if and only if $X$ and $Y$ are independent. The empirical version of covariance $V$ is applied to test the hypothesis. Specifically, with observations $\left \{X_{i}\right \}_{i=1}^{n}$ and $\left \{Y_{i}\right \}_{i=1}^{n}$, the empirical dCov is developed as the square root of


(2) \begin{align*}& \mathcal{V}_{n}^{2}(X, Y)=\frac{1}{n^{2}} \sum_{k, l=1}^{n} A_{k l} B_{k l},\end{align*}

where matrices $A$ and $B$ are determined by


(3) \begin{align*}& \begin{gathered} A_{k l}=a_{k l}-\bar{a}_{k \cdot}-\bar{a}_{\cdot l}+\bar{a}_{\cdot..}\\ B_{k l}=b_{k l}-\bar{b}_{k \cdot}-\bar{b}_{\cdot l}+\bar{b}_{...}, \quad k, l=1, \ldots, n \end{gathered}\end{align*}

with $a$ and $b$ representing the pairwise Euclidean distance matrices for the sets $\left \{X_{i}\right \}_{i=1}^{n}$ and $\left \{Y_{i}\right \}_{i=1}^{n}$, respectively, and


\begin{align*} &\bar{a}_{k \cdot}=\frac{1}{n} \sum_{l=1}^n a_{k l}, \quad \bar{b}_{k \cdot}=\frac{1}{n} \sum_{l=1}^n b_{k l}\end{align*}

\begin{align*} &\bar{a}_{\cdot l},=\frac{1}{n} \sum_{k=1}^n a_{k l}, \quad \bar{b}_{\cdot l},=\frac{1}{n} \sum_{k=1}^n b_{k l}\end{align*}

\begin{align*} &\bar{a}_{..}=\frac{1}{n^2} \sum_{k, l=1}^n a_{k l},\quad \bar{b}_{..}=\frac{1}{n^2} \sum_{k, l=1}^n b_{k l}\end{align*}

The empirical dCor is defined as the square root of


\begin{align*} & \mathcal{R}_n^2(\mathbf{X}, \mathbf{Y})= \begin{cases}\frac{\mathcal{V}_n^2(\mathbf{X}, \mathbf{Y})}{\sqrt{\mathcal{V}_n^2(\mathbf{X}) \mathcal{V}_n^2(\mathbf{Y})}}, & \mathcal{V}_n^2(\mathbf{X}) \mathcal{V}_n^2(\mathbf{Y})>0 \\ 0, & \mathcal{V}_n^2(\mathbf{X}) \mathcal{V}_n^2(\mathbf{Y})=0\end{cases} \end{align*}

and the $P$-value for the dCor association is derived via a permutation test [19]. Specifically, permutation tests were conducted on the gene expression data. First, we computed the dCor between the original gene expression datasets to establish an observed value. The gene expression values across samples are randomly shuffled while keeping the spatial locations fixed to generate the permutation distribution. This process breaks any inherent structure between gene expression and spatial locations, allowing us to assess the significance of the observed dCor under the null hypothesis of no association. The dCor is recalculated for each permutation using the permuted gene expression data. The observed dCor value was then compared to the permutation distribution. The $P$-value was computed as the proportion of permuted dCor values equal to or greater than the observed dCor, providing a measure of the statistical significance of the observed association.

Regarding the effectiveness of dCov for small sample sizes, such as less than 100, it is known that dCov is sensitive to sample size, similar to other statistical measures. Studies, including the original paper, indicate that dCov can still be effective with small sample sizes, but the power of the test increases with larger samples. For very small samples, the variability of the dCov statistic might increase, potentially affecting its reliability. From the computational aspect, the statistic $nV_{n}^{2}$ has $O(n^{2})$ time and space complexity [20].

### Testing between pathway gene expression and location

**Figure 1:**
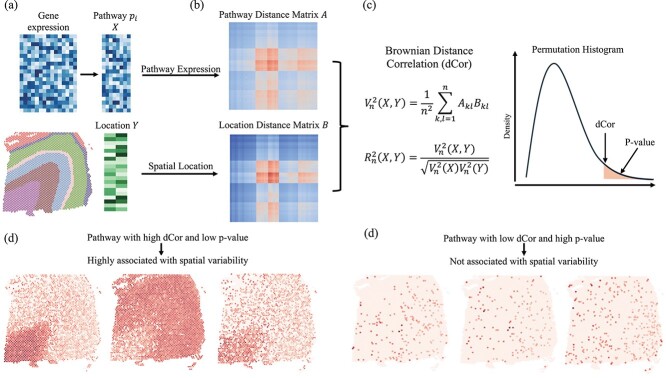
Schematic overview of analyzing the association between pathway expression and spatial location. (a) For a spatial transcriptomic dataset, the matrix X contains genes involved in pathway $p_{i}$, and the location matrix $Y$ is the spatial coordinate. (b) The Pathway distance matrix $A$ and location distance matrix $B$ are calculated based on matrix $X$ and $Y$, respectively. (c) The Brownian dCov $V_{n}(X,Y)$ and Brownian dCor $R_{n}(X,Y)$ are estimated using matrix $A$ and $B$. The $P$-value is estimated by permutation test. (d) Example heatmaps of genes in pathways with high dCor and significant $P$-value, indicating the pathway is highly associated with spatial location. (e) Example heatmaps of genes in pathways with low dCor and high $P$-value, indicating the pathway is not associated with spatial location.

We design a methodology based on the Brownian dCov test to explore the relationship between pathway gene expression and spatial location. Pathways with high dCor and significant $P$-values are recognized to be associated with spatial variation. Such analysis aids in our comprehension of regional variations in tissue functionality from a biological perspective. Figure 1 depicts the workflow and examples.

For spatial transcriptomic data with gene expression matrix $G \in \mathbb{R}^{n \times r}$ and spatial location matrix $L \in \mathbb{R}^{n \times 2}$, our initial step is pathway information retrieval. We utilize the Gene Ontology (GO) database [21], a comprehensive resource that annotates genes with their associated biological processes. From this database, we focus on biological processes that encompass total gene count ranging from 12 to 100, i.e. biological processes that are specific enough but not too small.

We denote the $m$ selected biological processes as $P = (P_{1}, P_{2},..., P_{m})$. For any $i \in [1,2,...,m]$, we denote the process $P_{i}$ encompasses $r_{i}$ genes. The list of associated gene names is represented as $P_{i} = [g_{i_{1}}, g_{i_{2}},..., g_{i_{r_{i}}}]$. With the gene list $P_{i}$, we define $G_{P_{i}}$ as a sub-matrix of the gene expression matrix $G$, containing only the genes associated with the $i$-th biological process $P_{i}$ shown in Fig. 1(a).

Given the sub-matrix $G_{P_{i}} \in \mathbb{R}^{n \times r_{i}}$, corresponding to biological process $P_{i}$, and the spatial location matrix $Y \in \mathbb{R}^{n \times 2}$, we can compute the pathway distance matrix $A$ and the spatial distance matrix $B$, both of which are of dimension $\mathbb{R}^{n \times n}$. These matrices represent the estimated spot-wise distances, with $A$ derived from gene expression data and $B$ reflecting the distances between spatial coordinates, respectively, shown in Fig. 1(b).

Subsequently, we utilize the Brownian dCor score to evaluate the spatial association, as depicted in the formula within Fig. 1(c). The $P$-value is determined through a permutation test to assess the statistical significance of this association. The highest test statistics are selected after examining all the $m$ pathways. The biological significance of these pathways is further validated by a comparative review of known tissue-specific functions and structures from the literature. Figure 1(d) illustrates an example heatmap of a pathway characterized by a high dCor and a significant $P$-value, suggesting a strong correlation with spatial variability. Conversely, Fig. 1(e) presents an opposite scenario. It is evident that one exhibits a distinct spatial pattern, whereas the other appears to follow a random distribution, which is consistent with the test results.

### Functional analysis of different regions within the same cell type

We next design an approach that utilizes the Brownian dCor in a supervised manner. In the following description, we illustrate the method using inner- and edge-cells. But the same approach can be used in other scenarios to study the behavioral difference of a pathway between two pre-specified cell groups.

**Figure 2:**
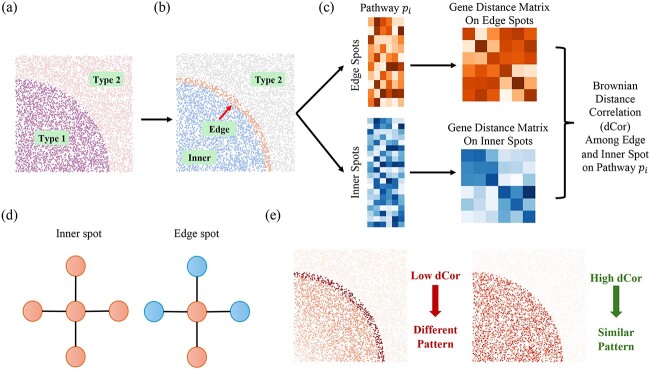
Framework for examining functional variability within the same cell types across different regions. (a) Illustration depicting a spatial region containing Type 1 and Type 2 cell regions. (b) Focus on Type 1 cell regions identifies distinct areas as either edge- or inner-regions. (c) For pathway $p_{i}$, two sub-expression matrices are delineated; one representing gene expressions at edge spots (orange) and the other at inner spots (blue). Subsequently, gene distance matrices for edge and inner spots are computed separately, facilitating the estimation of dCor. (d) Illustration of inner spot and edge spot. A spot is classified as an inner spot if all neighboring spots belong to the same cell type; otherwise, it is considered an edge spot. (e) Examples of different pathway patterns and similar pathway patterns between inner and edge regions.

To advance our understanding of the expression dynamics of pathways within diverse microenvironments, we focus on analyzing the biological functional disparities among different regions within the same cell type. Specifically, we divide spots of the same cell type into two distinct categories, edge and inner, utilizing available annotation and location information. This categorization facilitates a more comprehensive examination of functional variations, challenging the conventional notion of homogeneity based on cell type. The Brownian dCor is a particularly valuable metric for measuring the difference due to its versatility in accommodating random vectors across arbitrary dimensions and its non-negative properties. The dCor values close to zero indicate statistical independence. Figure 2 illustrates the analysis procedure and example.

In datasets annotated with cell type information, we classify cells based on spot location—either inner or edge—determined by the congruence of cell types between a spot and its neighboring regions. A spot is designated as “inner” if its cell type aligns with that of its immediate neighbors; conversely, it is categorized as an “edge” spot if there is a disparity in cell types.

For a given pathway $P_{i}$, we select relevant genes and construct the expression matrices for our regions of interest, i.e. the matrices for edge and inner expressions, symbolized by


\begin{align*} &G^{\prime}_{\text{in}} = [g_1^{\text{in}}, g_2^{\text{in}},...,g_{r_i}^{\text{in}}] \in \mathbb{R}^{n_{\text{in}} \times r_i}\end{align*}

\begin{align*} &G^{\prime}_{\text{edge}} = [g_1^{\text{edge}}, g_2^{\text{edge}},...,g_{r_{i}}^{\text{edge}}] \in \mathbb{R}^{n_{\text{edge}} \times r_i}\end{align*}

where $n_{\text{edge}}$ and $n_{\text{in}}$ denote the number of cells in the edge and inner areas, while $r_{i}$ represents the number of genes within the pathway. We calculate the gene Euclidean distance matrices $(a^{\text{edge}}_{kl}) = (|g_{k}^{\text{edge}} - g_{l}^{\text{edge}}|)$ and gene distance matrix on inner spots $(b^{\text{in}}_{kl}) = (|g_{k}^{\text{in}} - g_{l}^{\text{in}}|), k,l = 1,2,...,r_{i}$. According to the formula 3, $A^{\text{edge}}\in \mathbb{R}^{{r_{i}} \times r_{i}}$ and $B^{\text{in}}\in \mathbb{R}^{{r_{i}} \times r_{i}}$ can be calculated and thus we have the dCov


\begin{align*} &{V}_n^2(G^{\prime}_{\text{edge}}, G^{\prime}_{\text{in}})=\frac{1}{r_{i}^2} \sum_{k, l=1}^{r_i} A^{\text{edge}}_{k l} B^{\text{in}}_{k l},\end{align*}

and the dCor ${R}_{n}^{2}(G^{\prime}_{\text{edge}}, G^{\prime}_{\text{in}})$ depicting the difference among the two region in pathway $P_{i}$. Upon evaluating all pathways, we identify those pathways whose correlation values are closest to zero. This highlights pathways whose expression in the inner region is statistically independent of their expression in the edge region, thereby shedding light on divergent expression patterns between inner and edge spots.

## Results

### Pathways related to spatial location

#### Simulation study

To compare the power of our method with other existing methods for identifying spatially variable pathways, we conducted a simulation study focused on scenarios where the signal of an individual gene might be weak but becomes more pronounced when combined with the overall pattern of the entire pathway. When an individual gene exhibits a pronounced spatial pattern, it is often easily detected and classified as an SVG by various methods. Our approach, however, focuses on identifying SVGs based on pathways, leveraging existing biological information to analyze from a functional perspective. This perspective enables us to understand spatial specificity from a functional pathway angle. It enhances our ability to capture signals that might be overlooked when analyzing single genes. We compared our method with several popular tools, including SpatialDE [6], SPARK-X [8], SOMDE [22], and Giotto [23]. In Giotto, SVGs are identified through statistical enrichment in spatial nearest neighbors using Fisher’s exact test on binarized expression data. We tested two variations: one where genes are binarized using k-means clustering ($k$=2), and another where binarization is based on Giotto rank.

**Figure 3:**
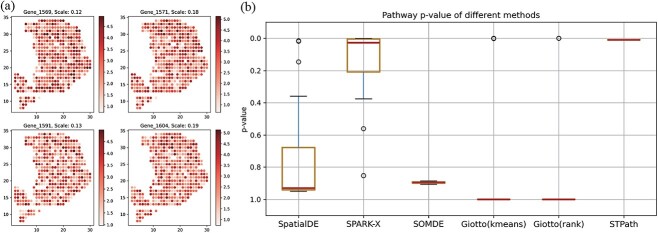
Comparison of our method with SpatialDE, SPARK-X, SOMDE, and Giotto in a simulation study. (a) Heatmaps of example genes with scale values ranging from 0.1 to 0.2. (b) Boxplot of pathway $P$-value across different methods.

To generate simulated data, we referred to the spatial structure of pancreatic ductal adenocarcinoma (PDAC) [24] and intentionally set the cancer region to have higher spatial expression features when generating spatial patterns. We set the number of genes to be 3000, with half of them being non-spatially variable genes (non-SVG) and the others being SVGs. For each non-SVG, we generated the gene expression values from a normal distribution with a mean of one and a standard deviation of one. For spatial variable genes, we used a combination of two components for the expression values. The first component was generated using a multivariate normal distribution, with mean values specified to reflect the spatial structure. In the cancer region, we set the means between 2 and 5, while for other regions, the means ranged from zero to two, highlighting the elevated expression in the cancer area. Covariance values were randomly assigned between 0 and 1, and we also ensured that the matrix diagonal elements were set to 1. The second component was derived from a random uniform distribution, producing expression values between 1 and 5 across the entire region. The final SVG expression was computed as a weighted sum of these two components, with the formula: $\text{SVG expression} = \text{scale} \times \text{expression}_{\text st} + (1-\text{scale}) \times \text{expression}_{\text rand}.$ The scale controls the visibility of the spatial pattern. The larger the scale, the more pronounced the pattern becomes. Figure 3(a) shows some example gene heatmaps with scale values ranging from 0.1 to 0.2, indicating a weak pattern for each single gene.

In our experiments, we created twenty pathways, each comprising one hundred genes. To mimic a more realistic pathway, we included fifty non-SVG genes and fifty SVG genes with scale values between 0.1 and 0.2 in each pathway. To evaluate the significance of each pathway using the gene-based methods, we combined the $P$-values of identified genes using the Cauchy combination test [25]. Specifically, to combines $k$ gene $P$-values $p_{1}$,...$p_{k}$ into a pathway $P$-value, we first calculate the $T$ statistic,


\begin{align*} & T=\frac{1}{n} \sum_{i=1}^n \tan \left(\pi\left(0.5-p_i\right)\right) \end{align*}

then the pathway $P$-value $p_{\text pathway}$ can be obtained using the Cauchy distribution:


\begin{align*} &p_{\text{pathway}} = 1- F(T),\end{align*}

where $F$ is the cumulative distribution of the Cauchy distribution. This method ensures a robust aggregation of individual $P$-values, yielding a comprehensive assessment of pathway-level significance. Although the spatial patterns of individual genes are not very pronounced and the pathway $P$-values from other methods are not significant, our method can still identify the spatial characteristics of pathways as shown in Fig. 3(b). This demonstrates the advantage of assessing spatial specificity from the perspective of the biological pathway expression. Discussion of the outliers observed in the histogram of pathway $P$-values is included in the Supplemental material.

#### Mouse olfactory bulb study

**Figure 4:**
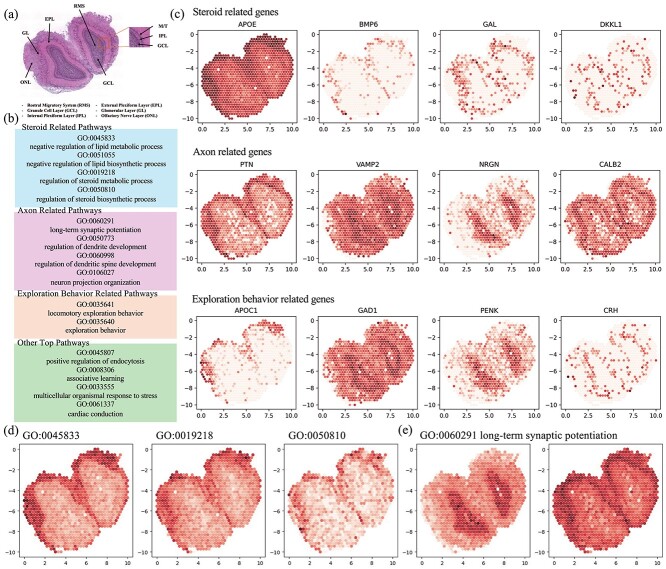
Application of STPath to MOB data. (a) Reconstructed image of H&E-stained, with sub-structure annotated. (b) Most significant pathways associated with spatial variation identified by STPath. We divide the pathways into four broad categories: steroid-related, axon-related, exploration behavior-related, and other pathways. (c) Example heatmaps of genes of top pathways related to steroid, axon, and exploration behavior. (d) Example heatmap of pathways of steroid-related pathways. (e) Heatmaps of long-term synaptic potentiation pathway.

We applied our method to Visium spatial transcriptomic data from the mouse olfactory bulb (MOB) (GSM4656181) with 10,000 permutations. Genes expressed in fewer than a pre-specified proportion of cells (twenty cells in this study) within the raw expression matrix were excluded. Subsequently, the expression counts were normalized and log-transformed using the SCANPY package [26]. Figure 4(a) displays the histological image of the dataset. Using annotations from other histologically marked images, regions within the image are outlined, including the Rostral Migratory Stream, Granule Cell Layer (GCL), Inner Plexiform Layer (IPL), External Plexiform Layer (EPL), Glomerular Layer (GL), and Outer Nuclear Layer (ONL). Figure 4(b) outlines the significant pathways associated with spatial variation identified by our method. These terms can be broadly categorized into pathways related to steroidogenesis, axonal structure and function, exploratory behavior, and other notable pathways.

One of the most different parts is lipids-related pathways, including negative regulation of lipid metabolic process (GO:00458 33), negative regulation of lipid biosynthetic process (GO:0051055), regulation of steroid metabolic process (GO:0019218), and regulation of biosynthetic process (GO:0050810). Lipids are crucial components of brain composition and function, playing an essential role in maintaining neuronal membrane structural integrity, which is vital for the normal functioning of neurons and the precise transmission of neural signals. The EPL primarily consists of dendrites from mitral cells and axons from peripheral neurons. At the same time, the GL is enriched with synaptic connections between olfactory neurons and secondary neurons [27, 28]. The olfactory nerve layer (ONL) contains olfactory nerve fibers extending from the nasal cavity, involved in the initial reception of olfactory signals [29]. Here, the regulation of lipid metabolism is particularly crucial for neuroprotection and preventing cellular damage caused by excessive lipid accumulation. Meanwhile, steroids, including sex hormones and corticosteroids, play critical roles in neuronal development, maintenance, and modulation [30]. The regulatory dynamics of steroid metabolism can fine-tune olfactory signaling, affecting stress responses and emotional regulation in the External Plexiform Layer. Studies suggest that steroid medications can improve olfactory nerve regeneration and reduce inflammation at injury sites [31].

Another set of essential terms identified relates to synaptic function and neuronal development. Long-term potentiation (LTP) is a form of enhanced communication between neurons closely associated with learning and memory. GO:0050773 and GO:0060998 refer to the regulation of dendrites and dendritic spines, respectively. These structures are essential for receiving input signals and forming synapses with other neurons. They participate in integrating and regulating signal output in the nervous system [32]. Neuronal Projection Organization (GO:0106027) involves the extension and branching of neuronal axons, facilitating communication within the olfactory bulb and potentially influencing the pathways of olfactory information transmission [33]. In Fig. 4(c), we observed differential gene expression of these terms between the GCL and GL layers. The GCL, despite lacking synapses, plays a central role as an intermediary neuron in olfactory signal processing [34], with inputs from olfactory receptor neurons initially organized and processed in the olfactory bulb’s GL [35]. The exploratory behavior observed in the IPL and outer plexiform layer (EPL) (GO:0035641 and GO:0035640) may be related to the integration and processing of olfactory information within these regions. Mammals associate odors with punishment or reward so that odors can trigger behaviors [36].

We also include examples of pathway heatmaps in Fig. 4. Since genes within a pathway do not always exhibit spatial variability, we selected genes that display spatial patterns and plotted the mean gene expression values to represent each pathway. Some terms, such as steroid-related pathways, exhibit similar spatial patterns, as shown in Fig. 4(d). In contrast, other pathways, like long-term synaptic potentiation, display multiple patterns, as in Fig. 4(e). Our method is capable of identifying these spatially related pathways in various cases.

The three showcased pathways related to lipid metabolic processes and steroid metabolic processes exhibit high expression concentrated in the ONL region. This is because the ONL contains the axons of olfactory sensory neurons (OSNs), which transmit olfactory information from the olfactory epithelium to the olfactory bulb. Lipid metabolism is crucial for the formation of cell membranes and the transmission of signaling molecules. At the same time, steroids can influence the growth and differentiation of neurons [37, 38]. In the olfactory bulb, neurosteroids may affect the processing of olfactory signals and the adaptability of sensory neurons [39]. Additionally, the olfactory system has a unique capacity for lifelong regeneration. Due to the continuous renewal and regeneration of OSNs, these metabolic processes support the growth and repair of neural structures, ensuring the normal function of the olfactory system [40, 41].

The two pattern example shown in Fig. 4 is long-term synaptic potentiation (LTP), a long-lasting increase in synaptic efficacy following high-frequency stimulation of afferent fibers. LTP is considered a primary mechanism for memory encoding and typically occurs in multiple regions of the nervous system, including the hippocampus, cortex, and other areas associated with cognitive functions [42–44]. One area where high expression of LTP pathways is concentrated in the GCL of the olfactory bulb. In the cerebellum, granule cells are the most abundant type of neuron.

Granule cells play a crucial role in local circuits in the olfactory bulb. They can regulate the transmission and processing of olfactory information through local synaptic plasticity, including LTP. Research has shown that a set of excitatory synapses on adult granule cells exhibit LTP shortly after entering the olfactory bulb [45]. Another region with high expression of LTP pathways is the ONL, composed of axonal projections from OSNs. These axons extend from olfactory receptor cells in the olfactory epithelium to postsynaptic targets in the glomeruli of the olfactory bulb [46]. Olfactory neuron axons form synaptic connections with incoming neurons in the olfactory bulb. Studies on the plasticity changes in dendritic mitral cells of the olfactory bulb after repeated stimulation have found that after repeated synaptic activation, LTP occurs at the synapses between mitral cell dendrites and granule cells, contributing to olfactory memory [47].

Although the primary focus of STPath is to identify spatially variable pathways, it can also identify variable genes as a special case when the pathway size is set to one. We included a case study on the MOB dataset to demonstrate the application of our method with single genes and compared it with SpatialDE. We calculated the dCor between each gene and the spatial location, then selected the genes with the highest dCor values as SVGs identified by our method. Detailed settings and comparisons are provided in the Supplemental material. The results show that genes identified by STPath exhibit denser and more robust spatial expression patterns compared to those identified by SpatialDE.

#### PDAC study

**Figure 6:**
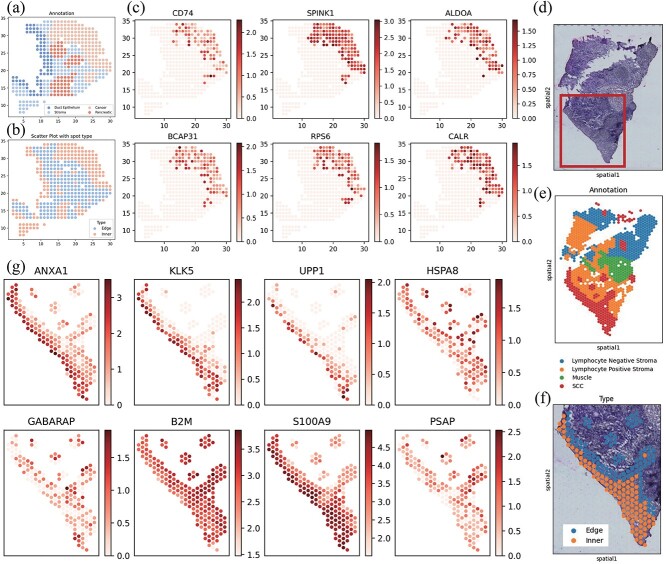
Functional analysis between inner and edge cancer spots on PDAC and OSCC datasets. (a) Annotated regions of PDAC: cancer, pancreatic, ductal, and stroma. (b) Depiction of inner and edge spots. (c) Heatmaps of genes related to GO:0042742, GO:0007276. (d) Reconstructed image of H&E-stained OSCC. (e) Annotated regions of OSCC: SCC, muscle, lymphocyte negative stroma, and lymphocyte positive stroma. (f) Depiction of inner and edge cancer spots of our interest cancer area. (g) Heatmaps of genes related to the most different pathways.

We applied our method to PDAC data [24]. The data processing was the same as the MOB dataset. Based on histological annotations, the data were categorized into several tissue regions, including cancer, duct epithelium, pancreatic, and stroma, as depicted in Fig. 6(a). Our analysis identified multiple significant pathways associated with spatial location variability. Given that we tested thousands of pathways, we applied multiple test correction to calculate the local FDR [48]. The results are presented in Table 1, which lists each pathway name, the count of related genes, the statistic (dCor), the $P$-value derived from our test, and the local FDR value.

**Table 1:** Most significant pathways associated with spatial variation on PDAC dataset.

**GOID**	**Term**	**n_gene**	**dCor**	** $P$ -value**	**local-fdr**
GO:0045104	intermediate filament cytoskeleton organization	25	0.598623	0.0001	0.000003
GO:0030307	positive regulation of cell growth	66	0.590806	0.0001	0.000003
GO:0008544	epidermis development	104	0.584986	0.0001	0.000003
GO:0006959	humoral immune response	84	0.566377	0.0001	0.000003
GO:0010927	cellular component assembly involved in morphogenesis	36	0.562825	0.0001	0.000003
GO:0042692	muscle cell differentiation	142	0.552465	0.0001	0.000003
GO:0010876	lipid localization	158	0.542856	0.0001	0.000003
GO:0048144	fibroblast proliferation	53	0.542380	0.0001	0.000003
GO:0050878	regulation of body fluid levels	150	0.542031	0.0001	0.000003
GO:0010951	negative regulation of endopeptidase activity	96	0.538407	0.0001	0.000003

One of the most significant pathways is the organization of the intermediate filament cytoskeleton. Intermediate filament plays a crucial role in providing essential structural support and contributing to cell integrity and signaling within the cytoskeleton. The fundamental process of cancer cell migration depends on the dynamic interplay between the cytoskeleton and cell surface receptors [49]. Simultaneously, tumor cells undergo metabolic adaptations to facilitate proliferation and metastasis, requiring the positive regulation of cell growth and cellular tissue architecture to undergo restructuring. Epithelial-to-mesenchymal transition induces a shift from an epithelial to a motile fibroblast-like morphology, which is pivotal within the tumor microenvironment of pancreatic ductal adenocarcinoma [50]. This transition has been demonstrated to play a significant role in the invasion and metastasis of PDAC [51]. Several other pathways involved in cell growth, differentiation, and development, well known for their association with cancer, are also found among the top 10 list, including positive regulation of cell growth, epidermis development, muscle cell differentiation *etc*. Another interesting biological process is the humoral immune response. PDAC has been studied for tumor-stroma interaction and cancer microenvironment [52].

**Figure 5:**
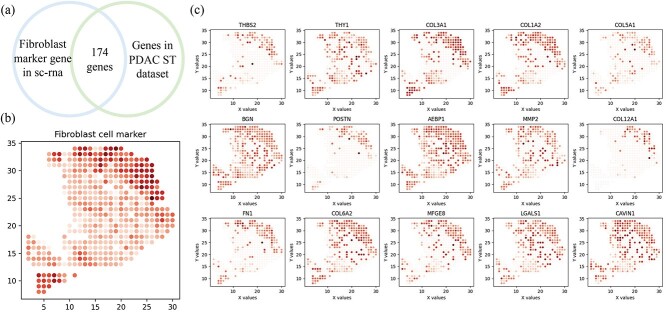
Study on fibroblast related marker genes. (a) The overlap gene region of the PDAC ST dataset and the fibroblast marker gene in sc-RNA data. (b) The heatmap of fibroblast cell related marker. (c) The gene heatmap of marker gene with spatial pattern.

In the original PDAC study [24], the authors identified specific genes related to inflammatory fibroblasts using single-cell RNA sequencing data. In our analysis, we found that the cell types in the single-cell data included fibroblasts but not specifically inflammatory fibroblasts. Since the original paper did not directly provide the list of the 90 genes they identified, we referred to the fibroblast marker gene list provided in the CARD tool using the same PDAC dataset [53]. The original marker list contained 224 genes, of which 174 genes were mapped to our spatial transcriptomics (ST) PDAC dataset (Fig. 5(a)). Since there is no GO term specifically named inflammatory fibroblast or fibroblast, we treated these 174 genes as a new composite marker for fibroblasts. The results of our STPath analysis regarding location variability are shown in Table 2. Our findings indicate that their expression is significantly associated with spatial location, exhibiting clear spatial specificity as shown in Fig. 5(b), with high expression concentrated in cancer regions and a small stroma area in the lower-left corner also showing high expression. Fig. 5(c) presents heatmaps of several genes with notable spatial specificity. In our main results, Table 1 highlights that we identified GO:0048144 (fibroblast proliferation) as related to spatial variation, which aligns with the findings on inflammatory fibroblasts in the original paper [24]. This evidence reinforces the credibility and significance of our methodology.

### Pathways related to cancer edge spots

#### Study on PDAC data

On PDAC data [24], we classified each spot as an edge spot or inner spot based on whether its neighboring spots belong to the same cell type. Figure 6(b) depicts this distinction, with inner spots colored red and edge spots colored blue. We ran functional analysis to compare inner- and edge-regions of cancer cells. Table 3 presents the top GO terms identified between inner- and edge-cancer cells by our method, highlighting the differences between inner and edge regions of the tumor.

The identified GO terms are associated with the development and progression of cancer, elucidating the complex behavior of cancer cells in environmental adaptation, signal transduction, cell migration, and immune evasion. One notable term is calcium ion transport, which involves TRPM7’s regulation of Ca$^{2+}$ levels, impacting calcium-permeable ion channels. This regulation can modify signaling pathways essential for survival, cell cycle progression, proliferation, growth, migration, invasion, and epithelial-mesenchymal transition (EMT), thereby influencing cell behavior and fostering tumor growth. Enhanced calcium ion transport around the tumor may promote cancer cell migration and invasion [54]. Other identified terms include anterograde trans-synaptic signaling. Research has demonstrated that dynamic interactions between cancer cells and neurons can enhance the invasiveness of PDAC, thereby supporting tumor occurrence, growth, and invasion [55].

Additionally, the regulation of cell growth is identified. TGF-$\beta $, a critical growth factor, plays a significant role in regulating cell proliferation, migration, and differentiation. During cancer progression, tumor cells may overcome the inhibitory effects of TGF-$\beta $ signaling through mutations, and TGF-$\beta $ can induce EMT, resulting in the loss of cell polarity and increased invasiveness of cancer cell [56]. Furthermore, Cancer cells at the tumor margin may undergo oxidative stress and cell death [57], which agrees with the identification of cellular responses to oxidative stress. Heatmaps of some representative genes associated with the top GO terms (GO:0042742 and GO:0007276) are illustrated in Fig. 6(c).

Considering the term inflammatory fibroblasts found in the original paper [24], we found no significant difference in fibroblast marker genes. As shown in Table 4, the dCor values were substantially greater than 0, indicating little difference between the two regions. In our analysis, Table 3 presents GO:0034599 (cellular response to oxidative stress), corroborating the original paper’s emphasis on the level of stress response in cancer regions. These pieces of evidence collectively support the validity and relevance of our approach.

**Table 2:** Result of testing whether the fibroblasts cell marker has spatial variation

**Term**	**Size**	** $P$ -value**	**dCor**
fibroblasts cell marker	174	0.0001	0.46684

**Table 3:** Top GO terms with divergent behavior between edge and inner cancer spots in PDAC data.

**GOID**	**Term**	**dCor**
GO:0042742	defense response to bacterium	0
GO:0007276	gamete generation	0
GO:0034599	cellular response to oxidative stress	0
GO:0006816	calcium ion transport	0
GO:0071559	response to transforming growth factor beta	0
GO:0002443	leukocyte mediated immunity	0.083
GO:0046034	ATP metabolic process	0.119
GO:0042327	positive regulation of phosphorylation	0.125
GO:0046700	heterocycle catabolic process	0.142
GO:0098916	anterograde trans-synaptic signaling	0.162

**Table 4:** Result of testing the fibroblasts cell marker among PDAC inner- and edge-cancer region.

**Term**	**Size**	**dCor**
fibroblasts cell marker	174	0.823734

#### Study on OSCC data

We applied our method to the spatial transcriptomic data of HPV-negative Oral Squamous Cell Carcinoma data (OSCC) [58], measured using 10xGenomics Visium platform. The image of H&E-stained tissue is shown in Fig. 6(d), with detailed annotations of the regions in Fig. 6(e). These areas include Squamous Cell Carcinoma (SCC), Lymphocyte Negative Stroma, Lymphocyte Positive Stroma, and Muscle. Metastasis and invasion are the major causes of mortality in OSCC patients [59], so understanding the microenvironmental functions at the tumor edge area is particularly important. Our focus is on analyzing SCC tumor cells to investigate if there are significant biological function differences between the inner- and edge-region. The study area is highlighted in the red box in Fig. 6(d). Tumor spots are divided into inner and edge based on the type of surrounding cells, with inner spots in orange and edge spots in blue, as shown in Fig. 6(f). Table 5 presents the top four pathways with the most notable differences.

**Table 5:** Top GO terms differentiating edge and inner cancer spots (OSCC).

**GOID**	**Term**	**dCor**
GO:0007186	G protein-coupled receptor signaling pathway	0.113
GO:0071496	cellular response to external stimulus	0.248
GO:0010975	regulation of neuron projection development	0.276
GO:0048545	response to steroid hormone	0.291

A critical pathway identified is the G protein-coupled receptor signaling (GPCRs) pathway (GO:0007186), which can regulate cell proliferation, activity, and movement. The signaling pathway is key to tumor growth, angiogenesis, and metastasis. At the interface between the tumor and lymphocyte-negative stroma, malignant cells may utilize the normal function of GPCRs to evade immune surveillance, enhance nutrition and oxygenation, and infiltrate surrounding tissues [60, 61].

The cellular response to external stimuli (GO:0071496) includes reactions to various external factors, such as increased interstitial pressure, changes in enzymatic activities, and pH level shifts. An increase in invasiveness and metabolic activity within tumor tissues leads to localized hypoxia in the tumor microenvironment, thereby raising the levels of regulatory transcription factors, such as hypoxia-inducible factors at the tumor edges. This hypoxic stimulus might further inhibit central metabolic synthesis, enhancing invasiveness and anti-apoptotic capabilities. At the edges, tumor cells may need to respond more to stimuli from the immune system, including cytokines released by immune cells [62–64].

The regulation of neurite outgrowth in oncology might relate to the proliferation of perineural fibers around tumors, which could be particularly prominent at tumor edges (related to GO:0010975, regulation of neuron projection development). Perineural invasion includes direct tumor invasion into surrounding nerve sheaths and the secretion of neurotrophic factors by tumor cells to promote cancer cell invasion and neurite growth [65]. Up to 80% of OSCC exhibits perineural infiltration, often resulting in pain or even cranial neuropathies [66].

Additionally, the response to steroid hormone signals (GO:0048545), such as estrogens, could enhance the motility of cancer cells by participating in the EMT process, crucial for the tumor’s invasiveness, dissemination, and metastasis capabilities. Evidence suggests that OSCC cell proliferation and invasiveness significantly increase following $\beta $-estradiol stimulation [67].

## Discussion

In this work, we utilize the Brownian covariance test from R package ‘energy’ to test spatial variable pathway that bypasses the selection of SVGs, as well as allows nonlinear and complex relations. Both are based on the testing of statistical dependencies between random vectors. One test is to find spatially associated pathways in an unsupervised manner, and the other is to find pathways associated with predetermined regions in a supervised manner. They can generate easily interpretable biological results from spatial transcriptomics data. We validate the effectiveness of the GO terms we identified by referencing existing literature, which often includes experimental evidence. While popular tools like ChatGPT can offer some insights, their outputs can sometimes be misleading or lack accurate and validated associations. Thus, a data-driven approach, supplemented by literature validation, is essential. Unlike ChatGPT, which concludes based solely on existing statements and studies, our method emphasizes a data-driven approach capable of uncovering novel connections between diseases and pathways. We believe our approach is a valuable addition to the tools for analyzing spatial transcriptomics data.

**Key Points**

- Our framework in spatial transcriptomics shifts the analytical focus from selecting spatially variable genes to identifying and analyzing functional pathways directly associated with spatial variability. This is achieved using a modified Brownian distance covariance test to assess the heterogeneity of biological functions spatially.
- The new framework does not require gene selection or parameter setting, allowing it to uncover nonlinear and complex dependencies in biological data.
- Our method enhanced our understanding of tissue dynamics. These insights contribute to a deeper understanding of cellular coordination and tissue architecture, particularly in the context of cancer pathology.

## Supplementary Material

STPath_supplemantary_bbae543

## Data Availability

Our study uses publicly available datasets. Human PDAC dataset is available from GSE111672, adult mouse hippocampus is publicly available in the National Center for Biotechnology Information (NCBI) GSM4656181, and oral squamous cell carcinoma is publicly available from GSE208253. We provide the Python and R code with description in GitHub https://github.com/tianlq-prog/STpathway.
