# Supplementary material for: A robust statistical approach for finding informative spatially associated pathways
Source: Brief Bioinform. 2024 Oct 25;25(6):bbae543. doi: 10.1093/bib/bbae543 (PMC11503753; doi:10.1093/bib/bbae543)
Supplement: STPath_supplemantary_bbae543 [file stpath_supplemantary_bbae543.pdf]

Supplementary information for: A Robust Statistical Approach for Finding Informative Spatially Associated Pathways

Clustering Performance on PDAC data

To allow maximal flexibility, our method uses a high-dimensional nonlinear association test. To assess the capability of the genes contained in the top spatially associated pathways with regard to the separation of cell types, we set the gene count used for clustering to be 500/1000/1500, and performed K-means clustering with a cluster size of four on the PDAC data using genes in the top spatially associated pathways, and compared with top highly variable genes. The clustering outcomes are visualized in Supplemental Figure 1 and evaluated using the Adjusted Rank Index (ARI) and the Normalized Mutual Infomation (NMI) against the annotated cell types. Our evaluation demonstrates genes associated with top spatially associated pathways are very informative with regard to separating annotated regions in the data.

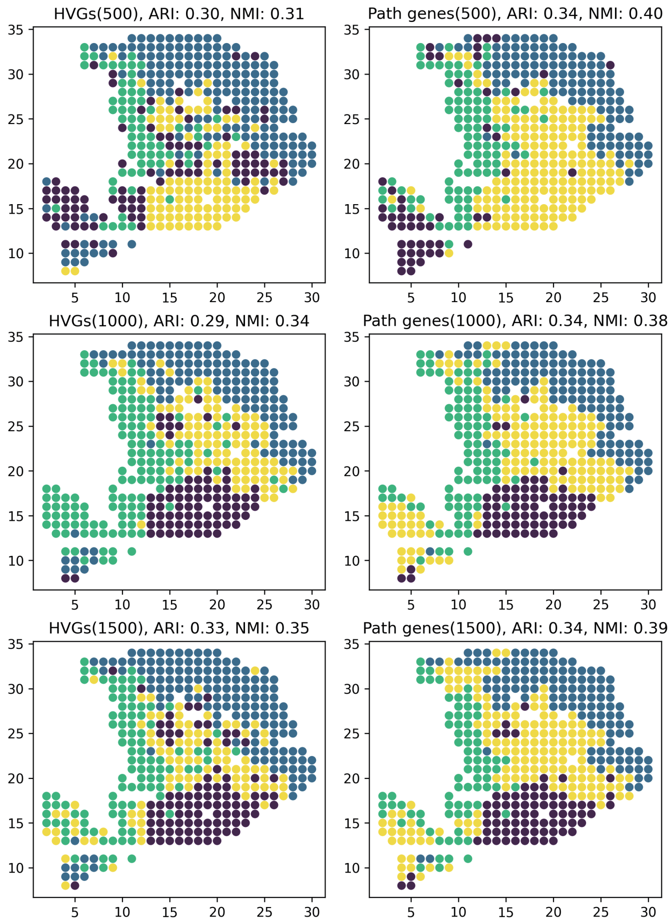

Supplemental Figure 1. The clustering result of PDAC data. The rows correspond to the gene size as 500, 1000 and 1500. The first column shows the clustering result using highly variable genes (HVGs), and the second column shows the result of top pathway genes.

Comparison of SpatialDE and one gene STPath

We discuss the effectiveness of our approach in identifying spatially variable genes. One of the key advantages of STPath is its ability to handle both single-gene and multi-gene pathways seamlessly, allowing for a more comprehensive analysis of spatial gene expression patterns.

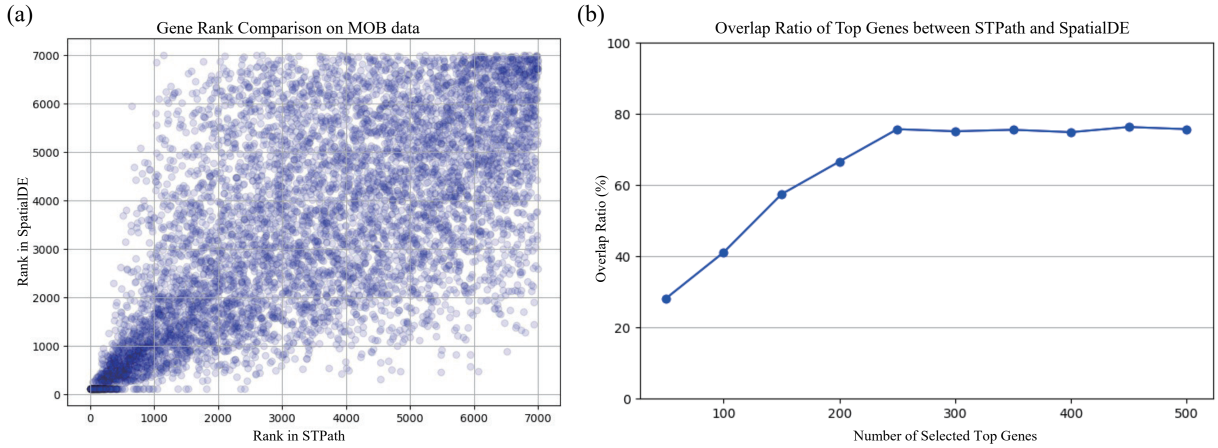

Supplemental Figure 2. (a) The scatter plot shows the ranks of genes identified by STPath and SptialDE on the MOB dataset. Each point represents a gene, with its rank in STPath on the x-axis and its rank in SpatialDE on the y-axis. (b) The overlap ratio of the top genes identified by STPath and SptialDE on the MOB dataset. The x-axis represents the number of selected top genes, ranging from 50 to 500 and the y-axis shows the overlap ratio.

We included a case study on the MOB Visium dataset to demonstrate the application of our method with single genes. Specifically, we applied STPath and SpatialDE for comparison. We calculated the dCor between each gene and the spatial location, then selected the genes with the highest dCor values as SVGs identified by our method. Supplemental Figure 2(a) shows the gene rankings in our method compared to SpatialDE, revealing a positive correlation between the two methods. To further explore the similarity between these methods, we calculated the overlap ratio between our method and SpatialDE for top gene sets of sizes 50, 100, 150, 200, 250, 300, 350, 400, 450, and 500. Supplemental Figure 2(b) shows that the overlap ratio remains close to 80% for gene sets larger than 200. However, the overlap is minimal for smaller gene sets, such as 50. To investigate the differences in the top selected genes, we present heatmaps of the top genes identified by STPath and SpatialDE SVGs in Supplemental Figure 3 and Supplemental Figure 4. Both methods identified genes with distinct spatial patterns. For instance, genes such as OLFR3 and TAGLN, which are high on the SpatialDE list, show very sparse expression, while genes identified by STPath exhibit denser expression patterns.

Study on fibroblast related marker genes on PDAC dataset

In the original PDAC study, the authors identified specific genes related to inflammatory fibroblasts using single-cell RNA sequencing data. In our analysis using the same dataset, we found that the cell types in the single-cell data included fibroblasts but not specifically inflammatory fibroblasts. Since the original paper did not directly provide the list of the 90 genes they identified, we referred to the fibroblast marker gene list provided in the CARD tool using the same PDAC dataset [1]. The original marker list contained 224 genes, of which 174 genes were mapped to our spatial transcriptomics (ST) PDAC dataset (Supplemental Figure 5(a)). Since there is no GO term specifically named inflammatory fibroblast or fibroblast, we treated these 174 genes as a new composite marker for fibroblasts. The results of our STPath analysis regarding location variability are shown in Table S**S1**. Our findings indicate that their expression is significantly associated with spatial location, exhibiting clear spatial specificity as shown in Supplemental Figure 5(b), with high expression concentrated in cancer regions and a small stroma area in the lower-left corner also showing high expression. Supplemental Figure 5(c) presents heatmaps of several genes with notable spatial specificity.

In our study of inner and edge cancer regions, we found no significant difference in fibroblast marker genes. As shown in Table S**S2**, the dCor values were substantially greater than 0, indicating little difference between the two regions. In our main results, Table 1 in the manuscript highlights that we identified GO:0048144 (fibroblast proliferation) as related to spatial variation, which aligns with the findings on inflammatory fibroblasts in Ref 28. Similarly, Table 2 presents GO:0034599 (cellular response to oxidative stress), corroborating the original paper [2]

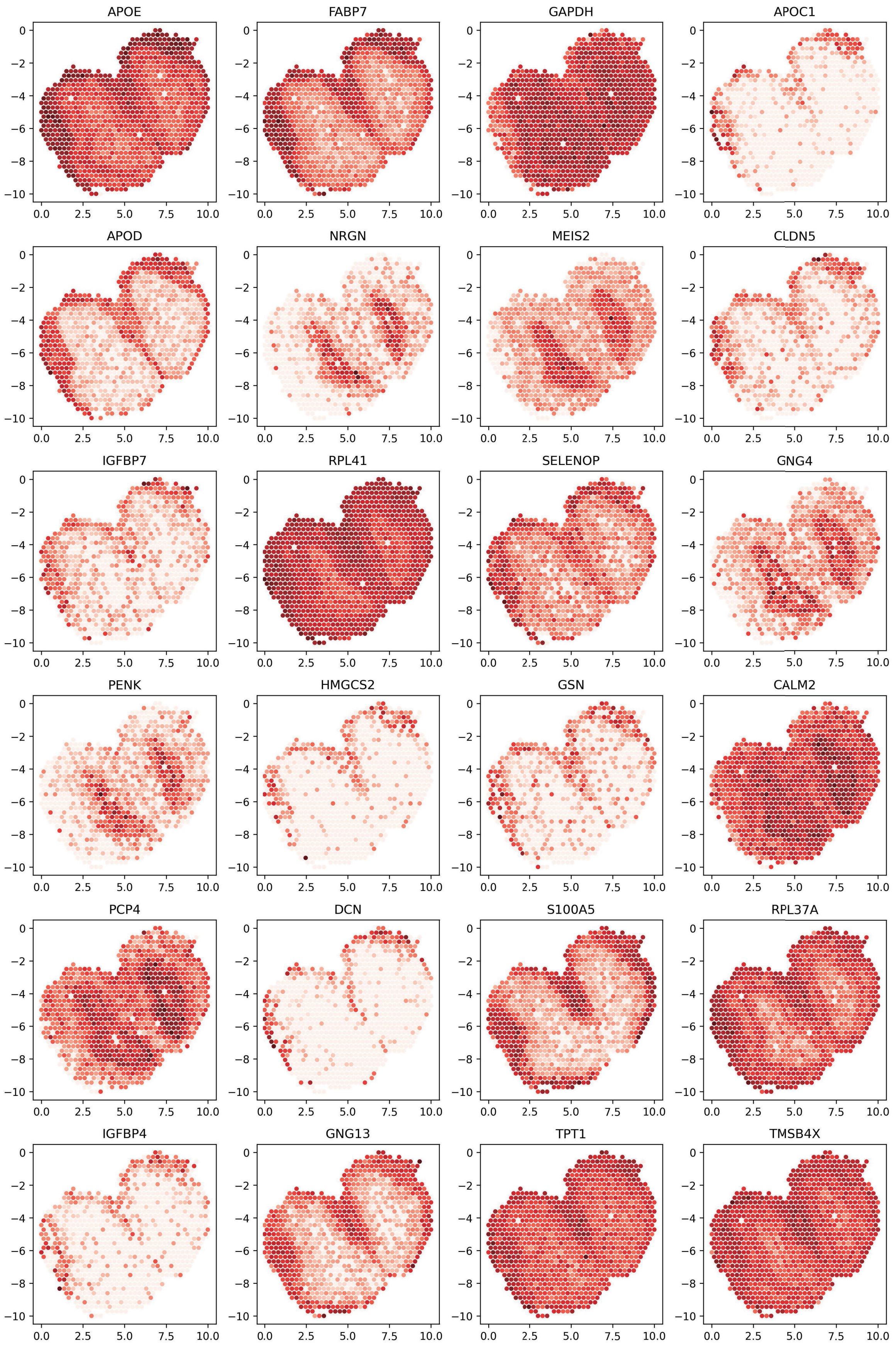

Supplemental Figure 3. Heatmap of the Top gene selected from STPath with highest dCor.

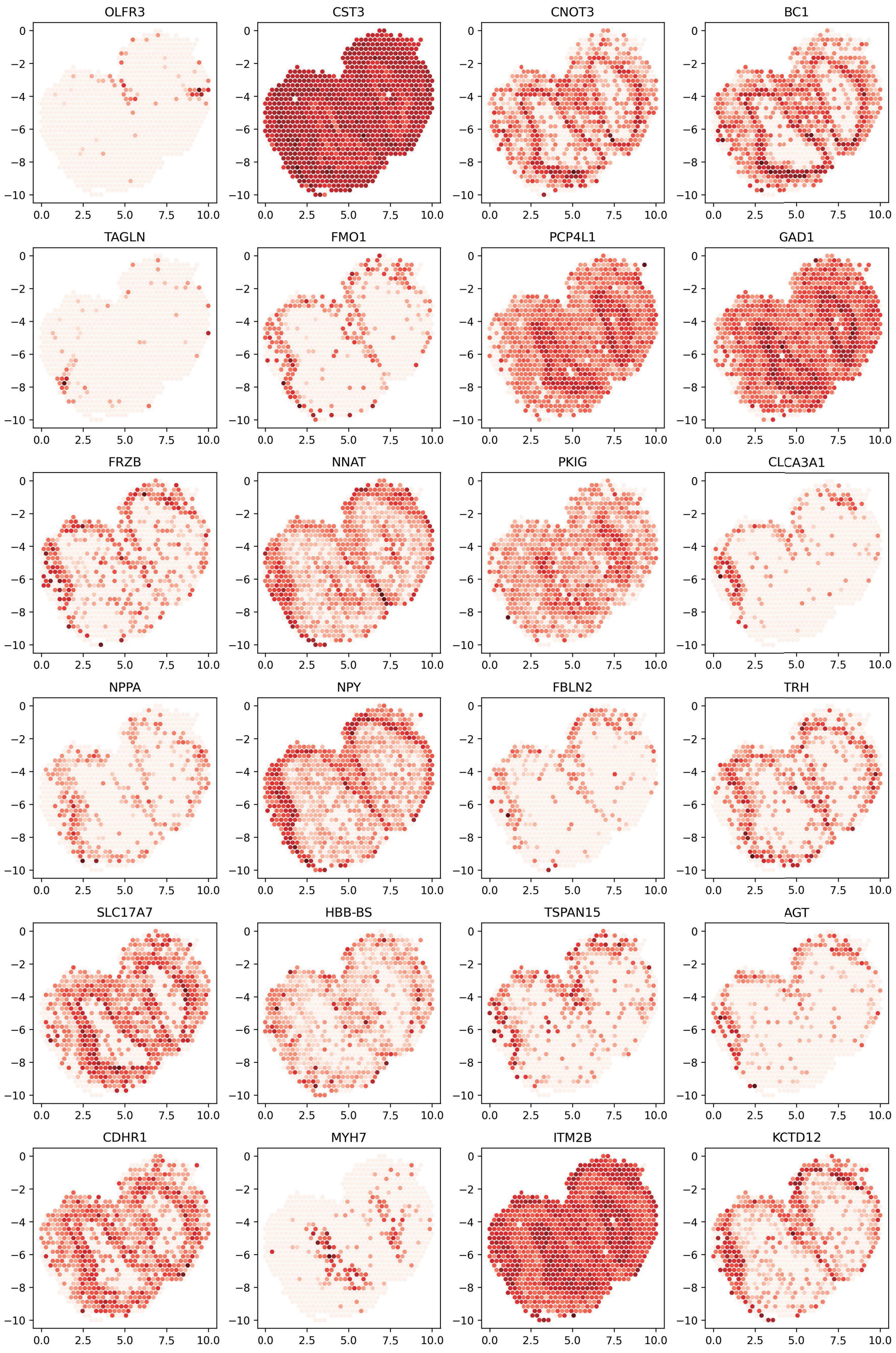

Supplemental Figure 4. Heatmap of the Top gene selected from SpatialDE.

emphasis on the level of stress-response in cancer regions. These pieces of evidence collectively support the validity and relevance of our approach.

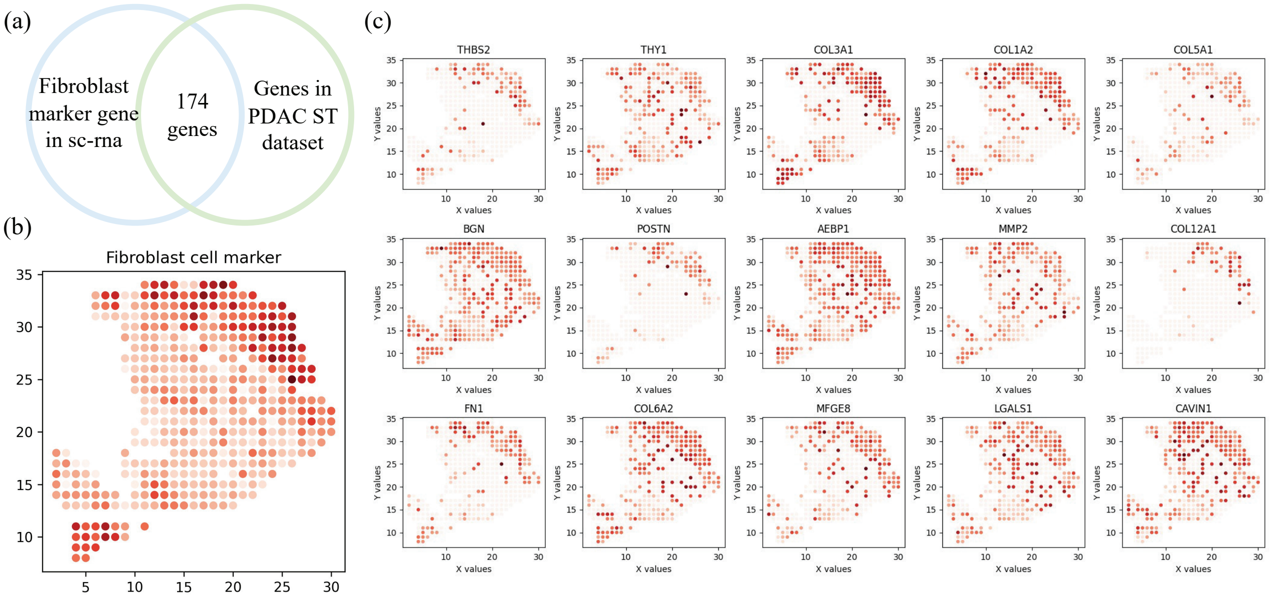

Supplemental Figure 5. Study on fibroblast related marker genes. (a) The overlap gene region of PDAC ST dataset and the fibroblast marker gene in sc-rna data. (b) The heatmap of fibroblast cell related marker. (c) The gene heatmap of marker gene with spatial pattern.

Table S1: dCor when testing whether the fibroblasts cell marker has spatial variation

Term	Size	P-value	dCor
fibroblasts cell marker	174	0.0001	0.46684

Table S2: dCor when testing the fibroblasts cell marker among inner- and edge- cancer region

Term	Size	dCor
fibroblasts cell marker	174	0.823734

Discussion about the outlier in simulation study

In our simulation study, we randomly selected related genes for each pathway. For methods other than STPath, we first calculated the p-value at the gene level and then aggregated these gene p-values using the Cauchy combination to assess pathway significance. Due to the inherent randomness in selecting different pathways during the simulation, outliers may appear across various methods.

Figure 6 illustrates the cases where outliers occur in the SpatialDE method (first line) and cases where outliers do not appear (second line). The figure shows that when a pathway contains a significant number of genes with highly significant p-values, the overall pathway significance increases, as shown in the first line of Figure 6. Conversely, when most genes in a pathway do not reach high significance levels, the pathway's significance decreases, as shown in the second line of Figure 6. Our method considers the entire pathway and all the genes it encompasses; thus, even in cases where other methods perform poorly, our approach can still clearly identify pathways with spatial variability.

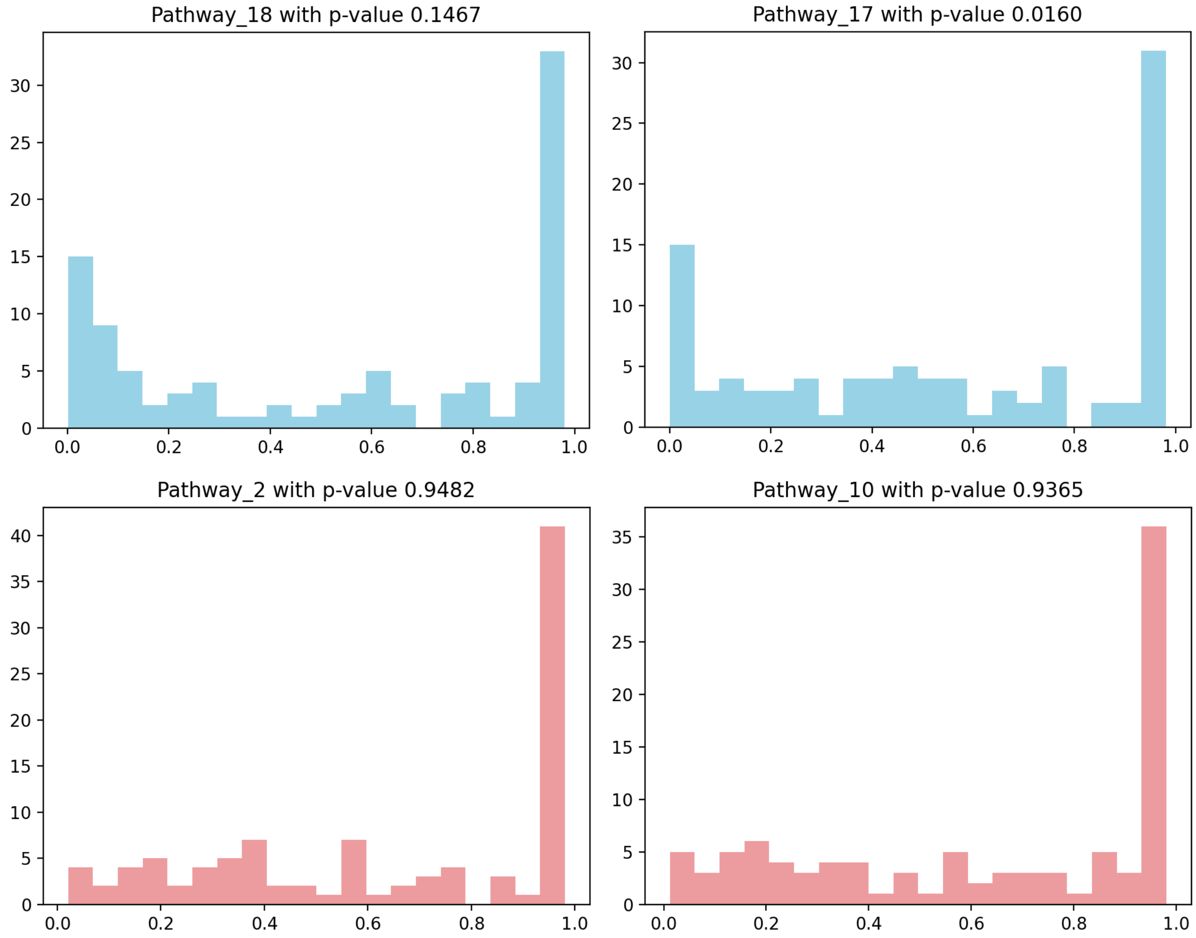

Supplemental Figure 6. Histogram of gene p-values across different pathways using SpatialDE. The first row (blue) represents outlier cases in the simulation study, characterized by a small pathway p-value. The second row (orange) depicts a general case with a large pathway p-value.

References

[1] Ying Ma and Xiang Zhou. Spatially informed cell-type deconvolution for spatial transcriptomics. *Nature biotechnology*, 40(9):1349–1359, 2022.

[2] Reuben Moncada, Dalia Barkley, Florian Wagner, Marta Chiodin, Joseph C Devlin, Maayan Baron, Cristina H Hajdu, Diane M Simeone, and Itai Yanai. Integrating microarray-based spatial transcriptomics and single-cell rna-seq reveals tissue architecture in pancreatic ductal adenocarcinomas. *Nature biotechnology*, 38(3):333–342, 2020.
